# Assessment of neonatal care in clinical training facilities in Kenya

**DOI:** 10.1136/archdischild-2014-306423

**Published:** 2014-08-19

**Authors:** Jalemba Aluvaala, Rachael Nyamai, Fred Were, Aggrey Wasunna, Rose Kosgei, Jamlick Karumbi, David Gathara, Mike English

**Affiliations:** 1 Ministry of Health, Government of Kenya, Nairobi, Kenya; 2 Department of Paediatrics and Child Health, University of Nairobi, Nairobi, Kenya; 3 KEMRI-Wellcome Trust Research Programme, Nairobi, Kenya; 4 Department of Obstetrics and Gynecology, University of Nairobi, Nairobi, Kenya; 5 Nuffield Department of Medicine & Department of Paediatrics, University of Oxford, Oxford, UK

**Keywords:** Neonatology, Health services research, Measurement, Evidence Based Medicine, Data Collection

## Abstract

**Objective:**

An audit of neonatal care services provided by clinical training centres was undertaken to identify areas requiring improvement as part of wider efforts to improve newborn survival in Kenya.

**Design:**

Cross-sectional study using indicators based on prior work in Kenya. Statistical analyses were descriptive with adjustment for clustering of data.

**Setting:**

Neonatal units of 22 public hospitals.

**Patients:**

Neonates aged <7 days.

**Main outcome measures:**

Quality of care was assessed in terms of availability of basic resources (principally equipment and drugs) and audit of case records for documentation of patient assessment and treatment at admission.

**Results:**

All hospitals had oxygen, 19/22 had resuscitation and phototherapy equipment, but some key resources were missing—for example kangaroo care was available in 14/22. Out of 1249 records, 56.9% (95% CI 36.2% to 77.6%) had a standard neonatal admission form. A median score of 0 out of 3 for symptoms of severe illness (IQR 0–3) and a median score of 6 out of 8 for signs of severe illness (IQR 4–7) were documented. Maternal HIV status was documented in 674/1249 (54%, 95% CI 41.9% to 66.1%) cases. Drug doses exceeded recommendations by >20% in prescriptions for penicillin (11.6%, 95% CI 3.4% to 32.8%) and gentamicin (18.5%, 95% CI 13.4% to 25%), respectively.

**Conclusions:**

Basic resources are generally available, but there are deficiencies in key areas. Poor documentation limits the use of routine data for quality improvement. Significant opportunities exist for improvement in service delivery and adherence to guidelines in hospitals providing professional training.

**What is already known on this topic**

- In Kenya, 60% of infant deaths and 40% of all under-5 deaths occur in the neonatal period.
- Clinical training facilities play a key role in effective delivery of essential neonatal interventions at a national scale.
- A previous study on a small number of Kenyan public hospitals suggested significant problems in provision of neonatal services.

**What this study adds**

- These are the most comprehensive data until now on routine neonatal care in a low-income African country.
- There is some improvement in the availability of basic resources and routine clinical practices.
- Errors in prescribing and provision of supportive care and poor data remain a challenge undermining practice and health service monitoring.

## Background

It is estimated that between 1990 and 2009, 79 million neonatal deaths occurred worldwide. Over 98% of these occurred in low-income and middle-income countries.^1^ In 2009, there were 42 013 neonatal deaths in Kenya,^1^ and as child mortality falls, the proportion of under-5 mortality due to neonatal deaths is rising. The most recent estimates indicate that 60% of infant deaths and 40% of all under-5 deaths occurred in the neonatal period,^2^ with this high neonatal mortality being a major reason why Kenya is not on track to achieve its fourth Millennium Development Goal target. The Ministry of Health has, therefore, started to prioritise interventions and investments to promote newborn (and maternal) health,^3^ basing its strategy on the essential newborn care package, including a number of low-cost, high-impact interventions.^4^ ^5^

In Kenya, health workers receive limited preservice instruction on neonatal care in their basic training, gaining most practical experience during clinical placements or internship in hospitals recognised as ‘internship training centres’. The knowledge and skills gained in such centres will likely therefore determine whether essential neonatal interventions are effectively delivered at a national scale. Unfortunately, a previous study on a small number of Kenyan public hospitals suggested significant problems in provision of neonatal services.^6^ ^7^ Therefore, in a partnership with Kenya's Ministry of Health, an assessment of neonatal care services provided by internship training centres was undertaken to identify areas requiring improvement as part of wider efforts to improve newborn and child survival.

## Methods

### Indicators

Indicators were based on prior work identifying national and international priorities^8^ and adapted from previous studies in Kenyan hospitals.^6^ ^7^ They focused on the Donabedian domains of structure (resources) and process.^6^ ^7^ ^9^ For structure, we checked for availability of core resources (see table 1) and if a recommended standard admission record form (newborn admission record, NAR) was in use. For process, we audited documentation of three key symptoms and eight key clinical signs of severe illness that are prioritised nationally,^10^ as they are associated with requirement for hospitalisation or referral.^11^ Dosages of prescribed antibiotics (as recorded on the treatment sheets) at admission were compared against those recommended in national guidelines. A margin of error of 20% above (overdose) and below (underdose) recommendations was allowed. Prescriptions of intravenous fluids and feeds were assessed in the same manner. Evidence of monitoring of vital signs, weight and fluids was defined as the presence of a chart(s) in which these were recorded at intervals. Mortality was the main outcome assessed.

**Table 1:** Availability of essential newborn care resources

Resources (n=22 hospitals)	Present n (%)
Ward organisation	
Most seriously ill babies are cared for in a section near nursing station	18 (82)
Isolation area in neonatal unit*	11 (50)
Hand hygiene	
Sink, clean running water and soap†	18 (82)
Alcohol hand rub	10 (46)
Emergency care	
Defined area for emergencies	13 (59)
Suction equipment working (n=20)‡	19 (95)
Bag valve mask set working (n=20)‡	19 (95)
Oxygen from any source available and working	22 (100)
Working pulse oximeter	4 (18)
Routine care	
Vitamin K (n=21)**§**	18 (86)
All babies adequately warmed	22 (100)
Special care/sick babies	
Working¶ phototherapy equipment (n=21)**§**	19 (91)
Benzylpenicillin	22 (100)
Gentamicin	18 (82)
Phenorbabitone injection	17 (77)
Kangaroo Mother Care (in any form)	14 (64)**
Paediatric burettes	12 (55)
Laboratory tests	
Blood glucose	22 (100)
Full haemogram	22 (100)
Bilirubin	19 (86)
Blood culture	10 (45)

Resource availability was assessed by direct observation (including checking drug stocks) by the researcher in the neonatal unit rather than by interviewing staff.

*Any of the following: designated isolation cot/incubator or a separate isolation room for separating sick (infected) babies from healthy ones.

**†**All three available.

**‡**These equipment (working or not) were available in 20/22 hospitals.

**§**Data missing for one hospital.

¶Working means the lights would turn on; irradiance was not measured.

**5/14 had a designated space for providing kangaroo care.

### Study design and population

This was a cluster survey of public hospitals providing internship training and the population of interest was admitted neonates aged <7 days.

### Sample size and sampling

At the time of survey, 40 public hospitals were recognised as providing internship training. The hospitals included were purposefully selected by the Ministry of Health to complement additional evaluation exercises, to ensure reasonable regional representation and with a sample size fixed at 22 sites because of budgetary constraints. Process of care was assessed by examining case records of admissions to the hospital area designated as the ‘newborn unit’. The number of cases per hospital was calculated to enable reporting of an observed proportion of 50% correct care across all hospitals with a precision (95% CI) of ±5%. To achieve this, assuming a coefficient of variation of 0.2 to account for clustering,^12^ we aimed to retrieve 60 case records per facility. Records were identified from the register of admissions starting from 31 May 2012 and going back through the register until 60 records were retrieved ensuring selection of records of those who had already been discharged or died.

### Data collection

Data collection was done over 4 weeks in July 2012. Survey staff comprised 22 Ministry of Health employees (nurses, records officers or clinical officers) with one drawn from each selected hospital. Staff underwent 1 week of training that included a pilot survey in a non-study hospital. Staff were subsequently divided into five teams (4–5 per team) that each visited 4–5 hospitals for 3–4 days.

Resource availability was assessed by walking through the neonatal units using a standard checklist.^13^ Availability was classified as universal (available in all 22 hospitals), mostly available (17–21), moderate (11–16) and low availability (0–10). Process of care and outcome data were entered directly into laptops using a data capture tool specifically designed for the survey in REDCap (Research Electronic Data Capture, a secure web-based application designed for research studies).^14^ A handbook of standard operating procedures was used in training and by all teams to guide all data entry.

Data were examined for errors in real time (in REDCap) and at the end of each day using STATA V.12 check files. Corrections were made by referring back to the source document under the supervision of the team leader. The clean data files from all sites were then uploaded into a central server.

### Statistical analysis

Availability of resources is presented as frequencies (and percentages) across the 22 hospitals. For process indicators, results are descriptive and where proportions were computed, the 95% CIs are adjusted for clustering at hospital level. Summary scores of symptoms and signs were constructed by allocating a score of 0 or 1 to each symptom or sign documented and summing these scores for each case record. We computed a median score and IQR for each hospital. To summarise across hospitals, the median of these median scores with the range across the 22 hospitals is reported. Outcomes are presented as mortality by birth weight.

## Results

### Hospital characteristics

We surveyed 22 hospitals of which 10 were administratively recognised as high-volume hospitals. The median number of deliveries per hospital in the month prior to the survey was 292 but ranged from 112 to 747. All hospitals had a neonatal unit and on the day of the survey, the median number of neonates in the units was 11 (range 1–47). Sixteen hospitals had a single paediatrician, six had two.

### Essential newborn care resources

At least one working source of oxygen was universally available (table 1). Bag valve masks, sinks, clean running water and soap were mostly available but paediatric intravenous fluid giving sets and a defined area for providing emergency care were moderately available. There was only low availability of alcohol hand rub or an area designated for kangaroo care. Stratification of resource availability by hospital category (normal/high volume) did not suggest any major differences (data not shown) except for the ability to undertake blood culture; available in 8/10 high-volume hospitals and 2/12 low-volume hospitals.

### Patient characteristics

A total of 1249 case records were examined (table 2). The most well-documented characteristic was mode of delivery with only 7% (82/1249) missing data, while the least documented was gestation by dates; 45% (561/1249) missing.

**Table 2:** Patient characteristics at admission of those included in process of care evaluation

Characteristics	Pooled data			Hospital-specific estimates	
	n	%	95% CI*	Median %	Range %
Sex (n=1088/1249, 87%)†					
Female	484	45	40 to 49	45	30–64
Male	604	56	51 to 60	55	36–70
Birth weight (n=1165/1249, 93%)†					
ELBW (<1000 g)	17	1.5	0.8 to 2.3	0.8	0–7.1
VLBW (1000–<1500 g)	118	10	6.9 to 15	7.1	1.7–46
LBW (1500–<2500 g)	370	32	29 to 35	33	19–46
Normal (2500–<4000 g)	607	52	48 to 57	54	21–68
LGA (≥4000 g)	53	4.6	3.3 to 63	3.6	0–13
Documented gestation by dates (n=688/1249, 55%)†					
Preterm	339	49	43 to 56	49	30–100
Term	344	50	44 to 56	50	0–68
Postdates	5	0.7	0.3 to 1.9	0	0–4
Mode of delivery (n=1167/1249, 93%)†					
SVD	767	66	60 to 71	66	42–96
Assisted vaginal	2	0.2	0.0004 to 0.7	0	0–1.8
Breech	26	2.2	1.2 to 4.3	0.8	0–11
Caesarean	372	32	27 to 38	32	3.9–51
Born before arrival (n=1055/1249, 84%)†	158	15	11 to 20	13	2.2–58

*Adjusted for clustering at hospital level.†n refers to the numerator equal to number of cases with data out of the total 1249 cases; the value for n becomes the item-specific denominator for each section.

ELBW, extremely low birth weight; LBW, low birth weight; LGA, large for gestational age; VLBW, very low birth weight; SVD, spontaneous vaginal delivery.

Available data showed 55% were male (604/1088, 95% CI 51% to 60%), 52% (607/1165, 95% CI 48% to 57%) had normal birth weight (2500–<4000 g) while one-third (32%, 370/1165, 95% CI 29% to 35%) were low birth weight (1500–<2500 g). There were similar numbers of preterm babies (<37 weeks gestation) 49% (339/688, 95% CI 43% to 56%) and term babies 50% (344/688, 95% CI 44% to 56%). Overall, most neonatal admissions followed spontaneous vaginal delivery, that is, 66% (767/1167, 95% CI 60% to 71%), but in individual hospitals caesarean section was as high as 51%.

**Figure 1:**
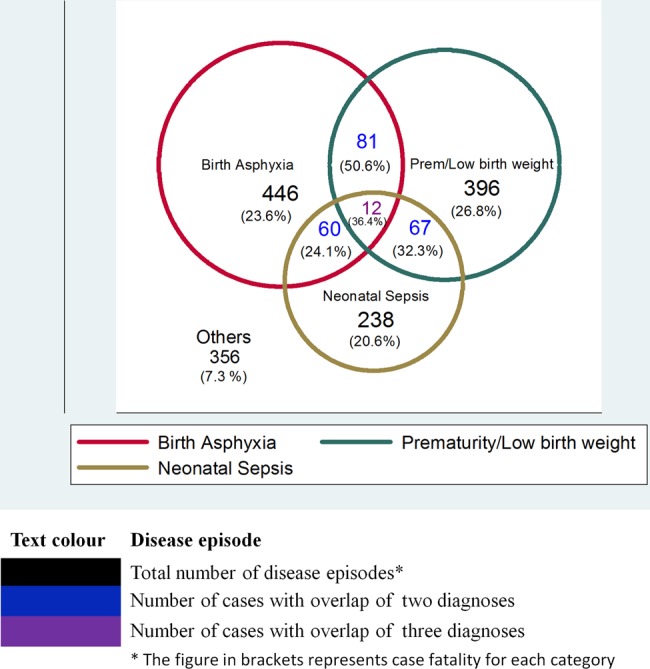
Top three disease episodes.

Three conditions accounted for the majority of disease episodes at admission: birth asphyxia 36% (446/1249, 95% CI 27% to 44%), prematurity/low birth weight 32% 396/1249, (95% CI 27% to 37%) and neonatal sepsis 19% (238/1249, 95% CI 14% to 25%). There were considerable overlaps in these three diagnoses (figure 1). Congenital anomalies were uncommon 8% (98/1249, 95% CI 2% to 14%), while the least commonly documented were meningitis, meconium aspiration and jaundice together accounting for <5%.

### Assessment

Overall, 57% (711/1249, 95% CI 36.2% to 77.6%) of the case files had an NAR form, but across hospitals ranged from 0% to 100% (table 3). Symptoms of severe illness were poorly documented with a median score of 0/3 overall (IQR 0–3), but six hospitals achieved a score of 3/3 (table 3) and all of them had the NAR in use. Signs of severe illness were better documented with an overall median of 6/8 signs documented (IQR 4–7). HIV exposure status was documented in only 54% (674/1249, 95% CI 41.9% to 66.1%). Among these, 12% of mothers were HIV positive (82/674, 95% CI 7.5% to 19.6%) but only two-thirds of their babies (54/82, 65.9%, 95% CI 46.9% to 80.8%) had a prescription for antiretroviral drugs in the case notes.

**Table 3:** Assessment, treatment and monitoring

	Pooled estimates			Hospital-specific estimates	
Indicator	n	%	Cluster adjusted 95% CI	Median %*	Range %†
1. NAR used (n=1249)	711	57	36 to 78	75	0–100
2. Symptoms and signs					
Symptom score (maximum 3)‡	0	0–3§	–	0	0–3
Sign score (maximum 8)¶	6	4–7§	–	5.8	0–8
3. Maternal HIV status					
Status documented	674	54	42 to 66	64	0–93
Positive	83/674	12	8 to 20	9.1	0–56
ARV for PMTCT prescribed for baby	54/82	66	47 to 81	–	–
4. Antibiotic prescription					
Benzylpenicillin dosage (n=778)					
Appropriate	649	83	66 to 93	93	0–100
Overdose	90	12	3.4 to 33	4.2	0–100**
Gentamicin dosage (n=761)					
Appropriate	473	62	51 to 73	67	7.7–91
Overdose	141	19	13 to 25	20	1.9–67
5. Supportive care					
Vitamin K prescribed (n=1213)	843	70	57 to 80	73	10–100
Appropriate dose of intravenous fluids (n=473)**††**	290	61	52 to 71	57	0–90
Appropriate amount of feeds (n=109)**††**	55	51	26 to 75	14	0–100
6. Medical review and monitoring					
Reviewed >24 h after admission (n=922)	20	2.2	1 to 4.8	0.8	0–21
Vital signs charted (n=1248)	970	78	68 to 87	85	8.3–100
Weight charted (n=1249)	510	41	25 to 56	39	0–100
Fluids monitored (n=559)‡‡	193	35	19 to 55	19	0–100

*Median of individual hospital median scores.

**†**Range of individual hospital median scores.

‡Difficulty feeding, convulsions and fits.

§IQR.

¶Temperature, bulging fontanelle, suck reflex/ability to feed, muscle tone, respiratory rate, severe indrawing, grunting and cyanosis.

**In the facility with 100% overdose, all penicillin doses were double the recommended.

**††**Treatment sheet with either intravenous fluids or supplementary feeds only for the first 24 h of life.

‡‡Five hundred and fifty-nine neonates had a fluid prescription on *any* day of life (473 of these on the *first* day).

ARV, antiretroviral; NAR, newborn admission record; PMTCT, preventing mother-to-child transmission.

### Treatment

About one in 10 of benzylpenicillin prescriptions was overdose (11.6%, 90/778, 95% CI 3.4% to 32.8%) (table 3) in contrast to almost one in five (18.5%, 141/761, 95% CI 13.4% to 25%) for gentamicin. Out of these 141 gentamicin overdose prescriptions, 87 (61.7%, 95% CI 50% to 72.4%) were 50% greater than the recommended dose. Birth weight was documented in 78 out of these 87 and the majority (68/78, 87.2%) were low birth weight (<2500 g).

### Supportive care, medical review and monitoring

From the pooled data, 70% (843/1213, 95% CI 56.9% to 79.7%) had Vitamin K prescribed (table 3) with two hospitals having 100% prescription rates. For those on intravenous fluids only on the first day of life, the pooled estimate had 61.3% (290/473, 95% CI 51.7% to 70.8%) with an appropriate volume. For those on feeds only, about one-half of the pooled estimates (51%, 95% CI 26% to 75%) had an appropriate volume. Time of first clinician doctors or paramedics known as clinical officers review after admission could be determined in 74% (922/1249), out of these 42% (383/922, 95% CI 27% to 58%) were seen within 6 h and 2% (20/922, 95 CI 1% to 5%) had no documented clinician review within the first 24 h. In general monitoring of weight, vital signs and fluids were poorly documented (table 3).

### Outcomes

Outcome by birth weight data was missing in 15% (184/1249) of records but where available the overall crude mortality was 17% (180/1065, 95% CI 11% to 24%) (table 4).The largest absolute number of deaths was among the normal birth weight (n=62). However, the highest case fatality was in the extremely low birthweight category (<1000 g) at 88% (14/16, 95% CI 58% to 97%). At individual hospital level, the highest mortality rate for newborn unit admissions within the sample of 60 cases was 46%. Of deaths, 61% (101/166, 95% CI 50% to 80%) occurred within the first 24 h after admission (table 4).

**Table 4:** Mortality by birth weight; extremely low birth weight (ELBW, <1000 g), very low birth weight (VLBW, 1000–<1500 g), low birth weight (LBW, 1500–<2500 g), normal (2500–<4000 g) and large for gestational age (LGA, ≥4000 g)

	Mortality				Time to death Time to death in days*
Birth weight	Pooled estimates		Hospital-specific estimates		
	n (%)	95% CI	Median (%)	Range (%)	Median (range)
ELBW (n=16)	14 (88)	58 to 97	100	0–100	1 (<1–54)
VLBW (n=100)	51 (51)	32 to 70	33	0–100	1 (<1–15)
LBW (n=340)	49 (14)	9 to 22	13	0–47	1 (<1–93)
Normal (n=559)	62 (11)	6.5 to 18	7.7	0–50	1 (<1–14)
LGA (n=50)	4 (8)	3.6 to 17	0	0–33	1.5 (<1–20)
Total (n=1065)†	180 (17)	11 to 24	16.4	0–46**‡**	1 (<1–93)

*Time to death from admission.

**†**In 15% of cases (184/1249), it was not possible to determine the outcome by birth weight (either due to missing outcome or birth weight data).

**‡**Four hospitals recorded no mortality, possibly representing a ‘retrieval’ bias in obtaining mortality files.

## Discussion

As neonatal mortality declines below 30/1000 (Kenya currently 31/1000), interventions delivered at facility level become increasingly important to achieve further declines.^15^ Each of the hospitals surveyed had a specific neonatal unit. They varied in size having between 2 and 15 working incubators and from 0 to 46 cots. Six hospitals were not able to allocate even one nurse specifically to their newborn unit.

All newborn units had at least one working source of oxygen and almost all were able to provide basic equipment for resuscitation and phototherapy. Key resources were missing in some hospitals, for instance, alcohol hand rub, bag valve mask sets and Kangaroo Mother Care (KMC). Although KMC is recommended for stable babies in national guidelines as it may reduce mortality and risk of sepsis and hypothermia,^16^ its implementation requires significant resources, including staff time. These resources are often not available likely explaining the challenges hospitals face in translating this policy into practice.

Resource limitations undermine the provision of basic neonatal care although there is improvement compared with a previous local survey.^7^ In that survey, half the hospitals did not offer phototherapy and less than half had phenorbabitone injection.^7^ Similar concerns have also been noted in Tanzania and Ghana,^17^ ^18^ Central Asia and Eastern Europe^19^ and Bangladesh.^20^ However, the specific nature of resource challenges differs across place underscoring the need for local knowledge such as from this survey to help plan improvement efforts.

Patient records provide a means to document and communicate information about patients and their care.^21^ They are also a vital source of data on workload, morbidity and mortality. Previous works on quality of neonatal care in Kenya and other countries have been severely limited by poor availability of records.^6^ ^17^ We retrieved a total of 1249 records but in some sites faced difficulties identifying admission records. Indeed, in four hospitals, no records of deaths were found raising the possibility that our results are affected by a form of response (or retrieval) bias. Such missing data undermine accurate reporting of patient characteristics and outcomes at scale. Despite this, our data remain the best current description of quality of routine neonatal service delivery for a country with over 1.25 million births per year.

Of concern is that almost 20% of gentamicin prescriptions were for an overdose and most were in preterm/low birthweight newborns with doses <50% above that recommended. Gentamicin is potentially ototoxic and nephrotoxic,^22^ ^23^ and drug level monitoring is not possible in any of the hospitals surveyed. Considerable variation in errors across hospitals is of great interest; for instance, the range of penicillin and gentamicin overdoses was 0%–100% and 1.9%–66.7%, respectively. Although standard guidelines are now more widely available in Kenya than previously,^10^ guidelines by themselves are insufficient to change practice. Thus, although there is evidence that neonatal prescribing is improving,^6^ ^9^ additional interventions that include regular assessment of quality of care may be required to promote good practices among early career clinicians at scale.^12^

The data available on outcomes are limited by missing data on birth weight, sex and particularly gestation. Available data suggest that many deaths occur early in admission and indicate very high case fatality in the extremely low birthweight babies; this may be a reflection of the lack of resources for more advanced care. However, the largest number of deaths occurred in normal weight births, suggesting significant opportunities to improve outcomes through improved basic perinatal and neonatal care. Better routine data in future may allow for analysis of the effect of quality of care on outcomes.

### Limitations

These results may not be representative of all hospital care for neonates in Kenya. Data are cross-sectional, from internship centres and based on observation of resources and record retrieval and review. Clinical practice in these hospitals is supervised by a paediatrician and thus may overestimate quality of care if results are generalised to the many hospitals with no paediatrician. In addition, we focused only on aspects of quality of care directly linked to implementation of guidelines. However, these are the most comprehensive data until now on routine neonatal care in a low-income African country.

## Conclusion

This audit shows improvement in the availability of basic resources and routine clinical practices. However, errors in prescribing and provision of supportive care and poor data remain a challenge undermining practice and health service monitoring. These data may indicate that such problems are more widespread in the region and we argue for specific efforts to promote quality care and monitor service delivery at scale as part of efforts to reduce newborn mortality.
